# Profiles of coagulase-positive and -negative staphylococci in retail pork: prevalence, antimicrobial resistance, enterotoxigenicity, and virulence factors

**DOI:** 10.5713/ajas.20.0660

**Published:** 2021-01-01

**Authors:** Gi Yong Lee, Soo-Jin Yang

**Affiliations:** 1 School of Bioresources and Bioscience, Chung-Ang University, Anseong 17546, Korea

**Keywords:** Pork, Staphylococci, Antimicrobial Resistance, Staphylococcal Enterotoxins (SEs), Virulence Factors

## Abstract

**Objective:**

The present study aimed to investigate the occurrence and species of coagulase-positive staphylococci (CoPS) and coagulase-negative staphylococci (CoNS) in retail pork meat samples collected during nationwide monitoring. The staphylococcal isolates were characterized for antimicrobial and zinc chloride resistance and enterotoxigenic potential.

**Methods:**

A total of 260 pre-packaged pork meat samples were collected from 35 retail markets in 8 provinces in Korea for isolation of staphylococci. Antimicrobial and zinc chloride resistance phenotypes, and genes associated with the resistance phenotypes were determined on the isolates. Furthermore, the presence and distribution of 19 staphylococcal enterotoxin (SE) genes and enterotoxin-like genes among the pork-associated staphylococci were determined by multiplex polymerase chain reaction-based assays using the specific primer sets.

**Results:**

A total of 29 staphylococcal strains (29/260, 11.1%) were isolated from samples of retail pork meat, 24 (83%) of which were CoNS. The four CoNS species identified were *S. saprophyticus* (n = 16, 55%), *S. sciuri* (n = 3, 10%), *S. warneri* (n = 3, 10%), and *S. epidermidis* (n = 2, 7%). Among the 29 isolates, four methicillin-resistant CoNS (MR-CoNS; three *S. sciuri* and one *S. epidermidis*) and one methicillin-resistant CoPS (MR-CoPS; one *S. aureus*) were identified. In addition, a relatively high level of tetracycline (TET) resistance (52%) was confirmed in CoNS, along with a predominant distribution of *tet*(K). The most prevalent SEs were *sep* (45%), and *sen* (28%), which were carried by 81% of *S. saprophyticus*.

**Conclusion:**

These findings suggest that CoNS, especially *S. saprophyticus* strains, in raw pork meat could be a potential risk factor for staphylococcal food poisoning (SFP), and therefore, requires further investigation to elucidate the role of SE*ls* in SFP and virulence of the pathogen. Our results also suggest that CoNS from raw pork meat may act as a source for transmission of antimicrobial resistance genes such as staphylococcal cassette chromosome *mec* and *tet*(K).

## INTRODUCTION

Staphylococci are commensal colonizers of the skin and mucous membranes of humans and other warm-blooded animals. Based on their ability to coagulate rabbit plasma, staphylococci have traditionally been grouped into coagulase-positive staphylococci (CoPS) or coagulase-negative staphylococci (CoNS). As a major human and animal pathogen CoPS, *S. aureus* can cause a variety of infections including superficial skin and soft tissue infections, osteomyelitis, and septicemia [1]. Although not usually life-threatening, staphylococcal food poisoning (SFP) is one of the most frequent food-borne diseases, and results from the ingestion of staphylococcal enterotoxins (SEs) that are secreted mainly by CoPS, especially *S. aureus* [1]. SFP is a noncontagious acute gastrointestinal disease caused shortly after ingestion of food contaminated with enterotoxin-producing staphylococci. Frequency of SFP is often associated with economic impact such as loss in productivity, financial burden for food industry, and public healthcare system [2]. Although a detailed pathogenic role of SE-producing CoNS in SFP has not been elucidated, recent studies have demonstrated that CoNS can also produce SEs and could be a potential cause of food poisoning [3]. Furthermore, classical SEs (SEA-SEE) and toxic shock syndrome toxin-1 (TSST-1) genes have been detected in CoNS isolated from human and animal clinical samples [3,4].

In addition to enterotoxigenicity, the incidence of SFP cases, especially those involving antimicrobial resistant (AMR) staphylococci such as methicillin-resistant staphylococci (MRS), has been increasing in recent years [4]. Methicillin-susceptible staphylococci (MSS) become MRS via acquisition of a staphylococcal cassette chromosome *mec* (SCC*mec*) containing the *mecA* gene. An increase in the carriage rate of these SCC*mec* elements in CoPS and CoNS from various food sources has also been demonstrated in several recent studies [5]. Wendlandt et al [6] also suggested that CoNS and MSS isolated from livestock farm environments represent a significant reservoir for transmission of AMR genes to MRS such as livestock-associated methicillin-resistant *S. aureus* (LA-MRSA). Therefore, enterotoxigenic staphylococci with enhanced resistance to antimicrobial drugs in food, especially livestock products, would raise significant public health concerns.

In swine production chains in European countries and Korea, one of the most frequent MRSA clones are CC398 (ST398 and ST541) LA-MRSA, which seem to be associated with the overuse of antibiotics in pig farms [7,8]. In addition to LA-MRSA, recent publications also suggested that CoNS and MSS in pig farm environments play an important role in transmission of antimicrobial resistance genes through horizontal gene transfer mechanism [5,9]. These observations raise a concern for transmission of antimicrobial resistant CoPS and CoNS via pork meat. However, the prevalence of CoNS in retail pork meat, their antimicrobial resistance profiles, and their enterotoxigenic characteristics are not well established.

The present study aimed to investigate the occurrence and species of CoPS and CoNS in retail pork meat samples collected during nationwide monitoring. In addition, antimicrobial resistance profiles, carriage of major antimicrobial resistance genes, and prevalence and distribution of SE genes and enterotoxin-like genes were determined in the pork-associated staphylococci. Moreover, susceptibilities to zinc chloride, urease activity, hemolytic activity, and proteolytic activity were examined to characterize the virulence phenotypes of the isolates.

## MATERIALS AND METHODS

### Sample collection

A total of 260 pre-packaged pork meat samples were collected from 35 retail markets in 8 provinces in Korea from July 2017 to May 2018: Gyeonggi (58 samples), Gangwon (42 samples), Chungcheong (55 samples), Jeolla (54 samples), and Gyeongsang (51 samples). Four or five retail markets from each province were randomly selected based on regional distribution. At least 3 to 4 different raw pork products including pork belly, loin, leg, blade shoulder, tenderloin, and arm shoulder were collected from each retail market. All meat samples were stored below 4°C in a container with ice packs and transported to the laboratory for isolation of staphylococci within 24 h of purchase.

### Isolation and identification of staphylococci

Isolation of staphylococci from pork meat samples was performed as described previously with minor modification [7,9]. Briefly, pork meat samples (25 g) were homogenized for 1 min in 225 mL buffered peptone water in a sterile stomacher bag (3M, St. Paul, MN, USA) using a stomacher (HUKO, Seoul, Korea). Next, 1 mL of the homogenized solution was added to 9 mL tryptic soy broth (TSB) supplemented with 10% NaCl and incubated at 37°C for 24 h. After pre-enrichment, 10 μL aliquots were streaked onto Baired-Parker agar (BPA) supplemented with egg yolk and potassium tellurite (Becton Dickinson [BD], Sparks, MD, USA) and incubated at 37°C for 24 to 48 h. Presumptive *S. aureus* with black colonies with a clear halo and/or staphylococcal colonies with gray/black color were all selected and up to 3 colonies of staphylococci from each sample were subcultured on BPA for further confirmation. Staphylococcal species identification was performed using both 16S RNA sequencing and the Vitek 2 system (bioMérieux, Marcy-l’Étoile, France). Genomic DNA samples of staphylococci were prepared using a Genemed kit (Seoul, Korea) according to the manufacturer’s recommendations. All the selected colonies were subjected to antimicrobial susceptibility assays and antimicrobial resistance gene detection assays.

### Determination of antimicrobial susceptibility

Antimicrobial susceptibility tests were performed on all the study strains according to the guidelines of Clinical and Laboratory Standards Institute (CLSI, 2017). Briefly, minimum inhibitory concentrations (MICs) to oxacillin and tetracycline (TET) were determined using a broth micro-dilution method or a standard E-test (bioMérieux, France). Susceptibilities to ampicillin (10 μg), cefoxitin (30 μg), gentamicin (30 μg), ciprofloxacin (5 μg), chloramphenicol (30 μg), clindamycin (2 μg), erythromycin (15 μg), sulfamethoxazole-trimethoprim (23.73 to 1.25 μg), quinopristin-dalfopristin (15 μg), rifampin (5 μg), fusidic acid (50 μg), and mupirocin (200 μg) were determined using a disc diffusion method on Mueller-Hinton agar plates (BD, USA). All the antimicrobial discs were purchased from BD. In addition to the antimicrobial susceptibility assays, MICs to zinc chloride (ranging from 0.125 to 32 mM) were determined using a standard agar dilution method on Mueller-Hinton agar (pH-adjusted to 5.5) as previously described [10].

### Detection of antimicrobial resistant genes and staphylococcal cassette chromosome mec typing

Staphylococcal strains displaying resistance to oxacillin, cefoxitin, and TET were examined for the presence of resistance genes. For methicillin-resistant strains, presence of *mecA* and SCC*mec* types were determined as previously described [11]. SCC*mec* types were determined according to the combination of chromosomal cassette recombinase (*ccr*) and methicillin resistance (*mec*) regions. TET resistant strains were tested for the presence of *tet*(K), *tet*(L), *tet*(M), *tet*(O), and *tet*(S) as previously described [12].

### Analyses of staphylococcal virulence factors

Urease activity was measured for 24 h with shaking (200 rpm). Urease activity was scored and classified into four groups according to intensity of color change: none (yellow), weak (orange), moderate (pink), or strong (purple).

Hemolytic activity was examined on 5% sheep blood agar after 24 h growth at 37°C. Presence of alpha-, beta-, or gamma-hemolytic activity was determined as previously described [13].

Proteolytic activity was assessed on skim milk agar (SMA) plates containing 10% SMA solution and 1.5% Bacto agar (BD Difco, USA) [14]. Briefly, 10 μL of the bacterial inoculum was dropped onto SMA plates and proteolytic activity was determined based on zones of clearing at 4°C and 37°C.

### Detection of staphylococcal enterotoxin genes

Carriage of SE genes in the 29 staphylococcal isolates was determined as previously described [2,15]. The presence of 5 classical SEs (*sea*, *seb*, *sec*, *sed*, and *see*) and 13 newer SEs (*seg*, *seh*, *sei*, *sej*, *sek*, *sel*, *sem*, *sen*, *seo*, *sep*, *seq*, *ser*, and *seu*) was examined using multiplex polymerase chain reaction (PCR) assays according to previous publications [15]. In addition to the SE genes, toxic shock syndrome toxin-1 (*tst1* gene) was assessed using specific PCR primers [15]. Genomic DNA samples from reference *S. aureus* strains (N315, FRI472, MW2, FRI913, and COL) were used as positive controls for each multiplex PCR [15].

## RESULTS

### Profiles of pork-associated staphylococci

A total of 29 staphylococcal strains (29/260, 11.1%) were isolated from 260 pork meat samples collected in Korea over the 11-month study period (Table 1). The most frequent pork-associated staphylococci observed in this investigation were *S. saprophyticus*, comprising 55% of the staphylococci isolated from the samples. As shown in Table 1, 5 CoPS and 24 CoNS (17.2% vs 82.8%) were identified among the 29 pork-associated staphylococci. While only two species of CoPS (*S. hyicus* and *S. aureus*) were detected in the samples, four different species of CoNS (*S. saprophyticus*, *S. sciuri*, *S. warneri*, and *S. epidermidis*) were identified.

The prevalence of staphylococci in the pork meat samples collected from different provinces is presented in Table 2. Interestingly, *S. saprophyticus* strains were isolated from all provinces, indicating that this is the most widespread staphylococcal species in pork meat samples in Korea. All the 29 staphylococci used in this study were isolated from different meat samples.

### Methicillin resistance among pork-associated staphylococci

Five MRS were identified among the 29 isolates (17%) collected from the pork meat samples (Table 1). These five isolates were all positive for *mecA* and displayed an OXA-resistant phenotype. SCC*mec* typing revealed that *S. aureus* (SA1), *S. epidermidis* (SE2), and *S. sciuri* (SS3) contained SCC*mec* II, SCC*mec* IV, and SCC*mec* V, respectively. Although two other *S. sciuri* strains (SS1 and SS2) were also positive for *mecA*, the SCC*mec* type remains undesignated because the *ccr* genes were not identified. The methicillin-resistant *S. aureus* strains were of sequence type (ST) 5, *agr* type II, and *spa* type t2460.

### Antimicrobial resistance profiles of staphylococci isolates

All the 29 isolates were susceptible to chloramphenicol, sulfamethoxazole-trimethoprim, and rifampin (Table 3). A multidrug resistance (MDR) phenotype was observed in four staphylococci (SA1, SH4, SS1, and SE2), which showed resistance to ≥3 different classes of antimicrobials. The highest level of antimicrobial resistance was observed for TET (15/29 staphylococci, 52%), and all 15 TET-resistant staphylococci harbored one or two *tet* genes with TET MICs of ≥32 μg/mL. Of note, 14/15 TET-resistant isolates (93%) carried the *tet*(K) resistance gene.

Zinc chloride resistance has been proposed to be associated with methicillin resistance in specific clones of *S. aureus* of animal origin [7,16,17]. However, as shown in Table 3, 14/29 (48%) staphylococci displayed resistance to zinc chloride (MICs ≥2 mM) regardless of methicillin resistance.

### Prevalence of staphylococcal enterotoxin genes

SEs are pyrogenic toxins and are involved in a significant number of human food poisoning diseases worldwide [2]. As shown in Table 4, all the staphylococcal isolates harbored one or more SE genes, except for three CoNS (SSP16, SS3, and SE1 strains), suggesting a widespread presence of SE genes among the CoPS and CoNS. A total of 11 different combinations of SE genes were identified among the 26 SE gene-positive staphylococci. Of the 18 SE genes analyzed, *sep* (13/29 isolates, 45%) and *sen* (8/29 isolates, 28%) were most frequently detected among the 29 staphylococci (Table 5). In particular, the *sep* and *sen* genes were present in 63% and 44% of *S. saprophyticucs* isolates, respectively. The SA1 strain (*S. aureus*) carried nine different SE genes and *tst1*, six of which are located within an enterotoxin gene cluster (*seg*, *sei*, *sem*, *sen*, *seo*, and *seu*). The presence of 6 enterotoxin genes (*sen*, *sep*, *seu*, *seq*, *seg*, and *sec*) identified in CoNS strains by PCR analyses were confirmed by sequencing PCR products (data not shown).

### Assessment of virulence factors

Phenotypic analyses of hemolytic activity in the 29 staphylococci revealed that all isolates were hemolytic: three *S. hyicus*, three *S. warneri*, and one *S. epidermidis* were alpha-hemolytic and all others were gamma-hemolytic (Table 4).

Urease activity was mainly associated with CoNS, with an overall positive rate of 88%. Of note, 100% of *S. saprophyticus*, *S. warneri*, and *S. epidermidis* strains showed strong urease activity.

Strong proteolytic activity was observed only in one *S. hyicus* and three *S. sciuri* strains at 37°C. None of the 29 staphylococci displayed proteolytic activity at 4°C.

## DISCUSSION

Although coagulase-positive *S. aureus* is the most common cause of SFP among staphylococcal species [1], recent studies have indicated that CoNS can produce SEs and become a potential cause of food poisoning. Staphylococci are normally present in the human and animal microbiota and are frequently found on the skin and in the upper respiratory tract of the host [18]. Thus, foods of animal origin such as milk, chicken, beef, and pork meats may represent a major source of SFP due to contamination with CoPS or CoNS during meat production. However, compared to *S. aureus*, only a few studies have addressed the prevalence and characteristics of non-*S. aureus* CoPS and CoNS in foods of animal origin.

In the present study, we investigated the prevalence of CoPS and CoNS in retail pork meat samples collected from July 2017 to May 2018 in Korea. The overall prevalence of staphylococci in the 260 pork meat samples was 11% (29 isolates/260 samples) and prevalence rates in five of the eight provinces ranged from 7% to 16%. Previous studies conducted in Nigeria, Poland, and Egypt reported prevalence rates of 40% to 91% for staphylococci in raw meats and meat products [4,19]. In particular, the prevalence of staphylococci in pork meat samples in Nigeria was 46%, which is significantly higher than the rate observed in the current study [19]. Consistent with prevalence rates reported in previous studies in Korea (0.4% to 19%), the prevalence of pork-associated *S. aureus* in the current study (0.4%, 1/260) was much lower than that reported for retail pork meat samples in the USA (16% to 67%) [20], Nigeria (54%), and China (18.6%) [21]. The present study reported that the proportion of CoNS observed among the 29 staphylococcal isolates was 83%, and this predominance of CoNS over CoPS is consistent with previous reports [4]. Four different CoNS species were identified and *S. saprophyticus* was detected with the highest incidence (16/29, 55%). In contrast, a predominance of *S. epidermidis* in raw pork meat was reported in a previous study from Nigeria [19]. These differences may have been caused by several factors such as geographical region, differences in pork production chains, and sampling/isolation methods. In addition, use of single methodology (enrichment in TSB supplemented with 10% NaCl and use of BPA) to isolate CoPS and CoNS would have affected the proportion of staphylococcal species presented in this study. However, these results suggest that CoNS isolates from raw pork meat could be a potential source of staphylococcal food contamination [4]. In addition, to the best of our knowledge, this is the first study to report the prevalence of CoPS as well as CoNS in retail pork meat collected nationwide in Korea.

MRS, especially methicillin-resistant *S. aureus* (MRSA), likely originated from CoNS by acquisition of SCC*mec* [22]. Methicillin-resistant CoNS have been isolated from a number of livestock and their products [3,4,23]. Consistent with these previous reports, four methicillin-resistant CoNS and one MRSA were isolated from raw pork meat samples in the current study. Although ST398 MRSA with SCC*mec* V has frequently been associated with livestock, particularly pigs [8], one strain of MRSA (SA1) isolated from raw pork was ST5 with SCC*mec* II. While one strain of *S. epidermidis* (SE2) and a strain of *S. sciuri* (SS3) was determined to harbor SCC*mec* IV and SCC*mec* V, two strains of *mecA*-positive *S. sciuri* (SS1 and SS2) could not be typed for SCC*mec*. Several studies have reported heterogeneity among SCC*mec* elements in MR-CoNS isolates [24]. In parallel with our results for *S. sciuri*, MR-*S. sciuri* carrying a non-typeable *ccr* gene was isolated from raw meat [25], indicating that a diverse pool of *ccr* genes and novel *ccr* types is present in *S. sciuri* isolates.

In addition to methicillin resistance, recent studies demonstrated that staphylococcal isolates from raw meat samples showed a TET resistance rate of 67% [26]. Similarly, a relatively high level of TET resistance (52%) was observed in staphylococci isolated in the current study, which is mainly attributed to TET-resistant *S. saprophyticus* isolates (11/16, 69%). TET resistance in staphylococci is mediated by acquisition of mobile *tet* genes, of which *tet*(K) has been most frequently associated with TET resistance in many CoNS of animal origin [3,18]. Consistent with previous observations [27], the present study found that 93% of TET-resistant staphylococci, and all the TET-resistant *S. saprophyticus* and *S. sciuri* isolates, carried *tet*(K).

A zinc resistance phenotype mediated by *czrC* within the SCC*mec* V element is typically associated with a high incidence of swine associated ST398 MRSA in European countries [10,28]. Recently, *czrC*-mediated zinc-resistance has also been reported in *S. aureus* strains isolated from meat products [29,30]. Unlike MRSA, data on the zinc resistance in non-*aureus* staphylococci (NAS) or CoNS are limited despite the possible co-selection of MRS using zinc in pig farms. However, the zinc-resistant gene *czrC* was identified in methicillin-resistant *S. hyicus* strains [31]. Moreover, frequent presence of *czrC* in methicillin resistant CoNS strains, such as *S. epidermidis*, *S. haemolyticus*, *S. hominis*, and *S. lentus* has been reported regardless of their SCC*mec* type [32]. Similarly, in the current study, 14/29 staphylococci displayed resistance to zinc chloride regardless of their methicillin or TET resistance, suggesting that co-selection of zinc resistance did not play a significant role in the high prevalence of methicillin- and TET-resistant CoNS strains used in this study. To the best of our knowledge, this is the first study in Korea that investigated the zinc resistance in NAS strains isolated from pork meat samples. Further studies are necessary to determine whether the use of zinc will be a major factor for co-selection of zinc and antimicrobial resistance in NAS or CoNS.

SEs were originally identified in *S. aureus* and are known to be responsible for SFP in humans. In addition to the 5 classical SE genes (*sea*, *seb*, *sec*, *sed*, and *see*), 19 newer SEs, and enterotoxin-like (SE*l*) peptides have been identified [2]. These SEs and SE*l*s are mainly associated with CoPS *S. aureus*-associated SFP worldwide [2]. However, recent studies reported CoNS such as *S. epidermidis*, *S. chromogenes*, *S. hyicus*, and *S. haemolyticus* isolates of human and animal origin carried SE genes [4,15]. In the present study, we found that CoNS isolated from pork meat samples more often possessed the newly described SEs and SE*l*s rather than the classical SEs. Moreover, our data showed that *sep* and *sen* were the most common SEs among the CoNS, especially in *S. saprophyticus* isolates. In contrast to the high prevalence of *sep* and *sen* in our CoNS, previous studies reported that *sec* was the most prevalent SE gene in CoNS isolates [33]. More recently, three classical SEs (*sea*, *seb*, and *sec*) were identified as the most frequent enterotoxins among CoNS isolated from bovine milk [23]. Although the role of SE*l*s in outbreaks of SFP is still controversial, the predominant presence of enterotoxigenic CoNS in raw pork meat could represent a potential food safety hazard. The SEs are synthesized and secreted by staphylococci throughout the exponential growth phase and during the transition from exponential to stationary growth phases [34,35]. Usually, the infective dose of enterotoxins needed to induce SFP in humans is only high nanogram to low microgram ranges. Synthesis of SEs is regulated by multiple factors such as bacterial cell density, changes in microenvironment, harsh growth conditions, and changes in bacterial cell membrane physiology [2,34,35]. These factors affect expression of enterotoxins through the alternative sigma factor (σ^B^), SarA, Agr quorum-sensing system, and SaeRS two-component regulatory system [2,34]. Unlike the classical SEs, information on the regulation of newer enterotoxins is limited even in coagulase-positive *S. aureus*. Therefore, further studies are necessary to characterize expression profiles of enterotoxin genes found in the CoNS, especially *sep* and *sen* genes in *S. saprophyticus* strains.

Staphylococci can produce secretary proteins such as pro teases and lipases that may have a negative effect on food preservation, causing a deterioration in quality and food spoilage [36]. Although protease-induced food spoilage has typically been associated with *Pseudomonas* spp. and psychrotrophic bacteria [36], pork meat derived coagulase-negative *S. hyicus* and *S. sciuri* strains showed strong protease activity at 37°C. In addition, strong urease activity, which is often associated with acute urinary tract infections, was only observed in CoNS species of *S. saprophyticus*, *S. warneri*, and *S. epidermidis*.

It should be recognized that the current study has several limitations. Our results were generated from a rather limited number of samples and staphylococcal isolates, which warrants future investigations with a larger number of various meat samples. In addition, complete genotypic characterization of the CoPS and CoNS for antimicrobial resistance, virulence, and synthesis of SEs in culture supernatants was not included in the current investigation.

## CONCLUSION

Our results demonstrate the following: i) a relatively high level of CoNS, especially *S. saprophyticus* species, are present in retail pork meat; ii) while the occurrence of MDR in pork-associated CoNS was low, a high level of TET resistance among CoNS was observed, along with a predominant possession of *tet*(K); and iii) a frequent presence of newly defined SE*l*s such as *sep* and *sen* were confirmed in CoNS species, especially in *S. saprophyticus* strains isolated from pork meat. Thus, in addition to CoPS, our results suggest that CoNS from raw pork meat could serve as a potential reservoir for antimicrobial resistance as well as enterotoxins typically associated with SFP. Future studies are also necessary to investigate prevalence and characteristics of CoPS and CoNS in other foods of animal origin such as milk, chicken, and beef meats.

## Figures and Tables

**Table 1:** Profiles of *Staphylococcus* spp. isolated from 260 pork meat samples

Staphylococcal strains	No. of methicillin-susceptible strains	No. of methicillin-resistant strains
CoPS (n = 5, 17%)		
*S. hyicus* (n = 4, 14%)	4	0
*S. aureus* (n = 1, 3%)	0	1 (SCC*mec*1) II)
CoNS (n = 24, 83%)		
*S. saprophyticus* (n = 16, 55%)	16	0
*S. sciuri* (n = 3, 10%)	0	3 (NT2), NT, SCC*mec* V)
*S. warneri* (n = 3, 10%)	3	0
*S. epidermidis* (n = 2, 7%)	1	1 (SCC*mec* IV)
Total 29 strains	24	5

CoPS, Coagulase-positive staphylococci; CoNS, Coagulase-negative staphylococci.

1) SCC*mec*, staphylococcal cassette chromosome *mec*.

2) NT, non-typeable SCC*mec*.

**Table 2:** Prevalence of CoPS and CoNS in pork meat samples

Staphylococcal strains	Number of staphylococcal isolates (%) in each province				

	Gyeonggi (58 samples)	Gangwon (42 samples)	Chungcheong (55 samples)	Jeolla (54 samples)	Gyeongsang (51 samples)
CoPS (n = 5)					
*S. aureus*		1 (2%)			
*S. hyicus*	1 (2%)		3 (5%)		
CoNS (n = 24)					
*S. saprophyticus*	2 (3%)	3 (7%)	4 (7%)	2 (4%)	5 (10%)
*S. sciuri*			1 (2%)	2 (4%)	
*S. warneri*					3 (6%)
*S. epidermidis*	2 (3%)				
Total 29 strains	5 (9%)	4 (10%)	8 (15%)	4 (7%)	8 (16%)

CoPS, coagulase-positive staphylococci; CoNS, coagulase-negative staphylococci.

**Table 3:** Antimicrobial resistance profiles, resistance genes, and zinc chloride resistance in CoPS and CoNS isolated from pork meat samples

Strain1)	Antimicrobial resistance profile2)	OXA MICs3) (μg/mL)	mecA	TET MICs4) (μg/mL)	*tet*5)	Zinc MICs6) (mM)	Distribution of SE and TSST-1 genes
SA1	AMP, CEF, CIP, CLI, ERY, GEN, OXA, TET	256	+	128	*tet*(M)	2	*seg, seh, sei, sel, sem, sen, seo, sep, seu, tst1*
SH1	SYN	0.25	-	0.064	-	1	*sej, sem, seo*
SH2	-	0.25	-	0.5	-	1	*sej, sem, seo*
SH3	-	0.25	-	0.5	-	4	*sej, sem, seo*
SH4	AMP, CIP, CLI, ERY, GEN, TET	0.125	-	64	*tet*(L), *tet*(K)	2	*sec, sej, ser*
SSP1	TET	0.5	-	16	*tet*(K), *tet*(S)	2	*sen*
SSP2	TET	0.5	-	64	*tet*(K)	2	*sen*
SSP3	-	0.5	-	0.5	-	2	*sen*
SSP4	TET	0.25	-	64	*tet*(K)	6	*sen*
SSP5	TET	0.25	-	64	*tet*(K)	6	*sen*
SSP6	TET	0.25	-	32	*tet*(K)	2	*sen, sep*
SSP7	TET	0.25	-	32	*tet*(K)	2	*sen, sep*
SSP8	TET	0.25	-	64	*tet*(K)	4	*sep, seu*
SSP9	TET	0.25	-	64	*tet*(K)	6	*seq*
SSP10	-	0.25	-	0.5	-	6	*sep*
SSP11	-	0.25	-	0.5	-	2	*seg, sep*
SSP12	TET	0.25	-	32	*tet*(K)	6	*seg, sep*
SSP13	-	0.25	-	0.5	-	6	*seg*
SSP14	-	0.125	-	0.5	-	4	*seg, sep*
SSP15	TET	0.125	-	32	*tet*(K)	2	*seg, sep*
SSP16	TET	0.25	-	64	*tet*(K)	2	-
SS1	CLI, FUS, OXA	1	+	0.25	-	4	*sep*
SS2	OXA	0.5	+	0.5	-	4	*sep*
SS3	FUS, OXA	0.5	+	0.5	-	4	-
SW1	-	0.125	-	0.25	-	2	*seq*
SW2	AMP, TET	0.25	-	32	*tet*(K)	4	*seq*
SW3	AMP, TET	0.25	-	32	*tet*(K)	4	*seq*
SE1	FUS	0.25	-	0.5	-	2	-
SE2	AMP, CEF, FUS, GEN, OXA	8	+	4	-	2	*seq*

CoPS, coagulase-positive staphylococci; CoNS, coagulase-negative staphylococci; SE, staphylococcal enterotoxin; TSST-1, toxic shock syndrome toxin-1.

1) SA, *S. aureus*; SH, *S. hyicus*; SSP, *S. saprophyticus*; SS, *S. sciuri*; SW, *S. warneri*; SE, *S. epidermidis*.

2) AMP, ampicillin; CEF, cefoxitin; CIP, ciprofloxacin; CLI, clindamycin; ERY, erythromycin; FUS, fusidic acid; GEN, gentamicin; OXA, oxacillin; SYN, quinupristin-dalfopristin; TET, tetracycline.

3) Oxacillin minimum inhibitory concentrations (MICs); CoPS >8 μg/mL, CoNS ≥0.5 μg/mL.

4) Tetracycline MICs ≥16 μg/mL indicate resistance.

5) Tetracycline resistance genes; *tet*(M), *tet*(L), *tet*(K), *tet*(O), and *tet*(S).

6) Values of >2 mM indicate resistance.

**Table 4:** Carriage of SE and TSST-1 genes and exoenzyme activities in CoPS and CoNS isolated from pork meat samples

Strain	Distribution of SE and TSST-1 genes	*egc*1)	Urease activity2)	Hemolytic activity	Proteolytic activity2)	

					4°C	37°C
SA1	*seg, seh, sei, sel, sem, sen, seo, sep, seu, tst1*	+	+	γ	-	-
SH1	*sej, sem, seo*	-	-	α	-	-
SH2	*sej, sem, seo*	-	-	α	-	-
SH3	*sej, sem, seo*	-	-	α	-	-
SH4	*sec, sej, ser*	-	-	α	-	+++
SSP1	*sen*	-	+++	γ	-	-
SSP2	*sen*	-	+++	γ	-	-
SSP3	*sen*	-	+++	γ	-	-
SSP4	*sen*	-	+++	γ	-	-
SSP5	*sen*	-	+++	γ	-	-
SSP6	*sen, sep*	-	+++	γ	-	-
SSP7	*sen, sep*	-	+++	γ	-	-
SSP8	*sep, seu*	-	+++	γ	-	-
SSP9	*seq*	-	+++	γ	-	-
SSP10	*sep*	-	+++	γ	-	-
SSP11	*seg, sep*	-	+++	γ	-	-
SSP12	*seg, sep*	-	+++	γ	-	-
SSP13	*seg*	-	+++	γ	-	-
SSP14	*seg, sep*	-	+++	γ	-	-
SSP15	*seg, sep*	-	+++	γ	-	-
SSP16	*-*		+++	γ	-	-
SS1	*sep*	-	-	γ	-	+++
SS2	*sep*	-	-	γ	-	+++
SS3	*-*		-	γ	-	+++
SW1	*seq*	-	+++	α	-	+
SW2	*seq*	-	+++	α	-	-
SW3	*seq*	-	+++	α	-	-
SE1	-		+++	γ	-	-
SE2	*seq*	-	+++	α	-	+

SE, staphylococcal enterotoxin; TSST-1, toxic shock syndrome toxin-1; CoPS, coagulase-positive staphylococci; CoNS, coagulase-negative staphylococci.

1) Screened for *egc* locus genes; *seg*, *sei*, *sem*, *sen*, *seo* and *seu*.

2) −, none; +, weak; ++, moderate; +++, strong.

**Table 5:** Carriage of SE genes in staphylococcal strains isolated from pork meat samples

Species (No. of isolates)	Number of SE gene-positive isolates																			

	SEs	*sea*	*seb*	*sec*	*sed*	*see*	*tst1*	*seg*	*seh*	*sei*	*sej*	*sek*	*sel*	*sem*	*sen*	*seo*	*sep*	*seq*	*ser*	*seu*
*S. aureus* (n = 1)	1						1	1	1	1			1	1	1	1	1			1
*S. hyicus* (n = 4)	4			1							4			3		3			1	
*S. saprophyticus* (n = 16)	6														7		10			1
*S. sciuri* (n = 3)	3									1							2			
*S. warneri* (n = 3)	3																	3		
*S. epidermidis* (n = 2)	1																	1		
Total	18			1			1	1	1	2	4		1	4	8	4	13	4	1	2

Number of isolates positive for at least one SEs.

SE, staphylococcal enterotoxin.

## References

[1] Li H, Andersen PS, Stegger M (2019). Antimicrobial resistance and virulence gene profiles of methicillin-resistant and -susceptible *Staphylococcus aureus* from food products in Denmark. Front Microbiol.

[2] Fisher EL, Otto M, Cheung GYC (2018). Basis of virulence in enterotoxin-mediated staphylococcal food poisoning. Front Microbiol.

[3] Pyzik E, Marek A, Stepien-Pysniak D, Urban-Chmiel R, Jarosz LS, Jagiello-Podebska I (2019). Detection of antibiotic resistance and classical enterotoxin genes in coagulase-negative staphylococci isolated from poultry in Poland. J Vet Res.

[4] Fijalkowski K, Peitler D, Karakulska J (2016). Staphylococci isolated from ready-to-eat meat - identification, antibiotic resistance and toxin gene profile. Int J Food Microbiol.

[5] Vanderhaeghen W, Vandendriessche S, Crombe F (2012). Species and staphylococcal cassette chromosome *mec* (SCC*mec*) diversity among methicillin-resistant non-*Staphylococcus aureus* staphylococci isolated from pigs. Vet Microbiol.

[6] Wendlandt S, Shen J, Kadlec K (2015). Multidrug resistance genes in staphylococci from animals that confer resistance to critically and highly important antimicrobial agents in human medicine. Trends Microbiol.

[7] Back SH, Eom HS, Lee HH, Lee GY, Park KT, Yang SJ (2020). Livestock-associated methicillin-resistant *Staphylococcus aureus* in Korea: antimicrobial resistance and molecular characteristics of LA-MRSA strains isolated from pigs, pig farmers, and farm environment. J Vet Sci.

[8] Price LB, Stegger M, Hasman H (2012). *Staphylococcus aureus* CC398: host adaptation and emergence of methicillin resistance in livestock. mBio.

[9] Eom HS, Back SH, Lee HH, Lee GY, Yang SJ (2019). Prevalence and characteristics of livestock-associated methicillin-susceptible *Staphylococcus aureus* in the pork production chain in Korea. J Vet Sci.

[10] Hau SJ, Frana T, Sun J, Davies PR, Nicholson TL (2017). Zinc resistance within swine-associated methicillin-resistant *Staphylococcus aureus* isolates in the United States is associated with multilocus sequence type lineage. Appl Environ Microbiol.

[11] Kondo Y, Ito T, Ma XX (2007). Combination of multiplex PCRs for staphylococcal cassette chromosome *mec* type assignment: rapid identification system for *mec*, *ccr*, and major differences in junkyard regions. Antimicrob Agents Chemother.

[12] Ng LK, Martin I, Alfa M, Mulvey M (2001). Multiplex PCR for the detection of tetracycline resistant genes. Mol Cell Probes.

[13] Clyne M, De Azavedo J, Carlson E, Arbuthnott J (1988). Production of gamma-hemolysin and lack of production of alpha-hemolysin by *Staphylococcus aureus* strains associated with toxic shock syndrome. J Clin Microbiol.

[14] Lee HS, Kwon M, Heo S, Kim MG, Kim GB (2017). Characterization of the biodiversity of the spoilage microbiota in chicken meat using next generation sequencing and culture dependent approach. Korean J Food Sci Anim Resour.

[15] Park JY, Fox LK, Seo KS (2011). Detection of classical and newly described staphylococcal superantigen genes in coagulase-negative staphylococci isolated from bovine intramammary infections. Vet Microbiol.

[16] Aarestrup FM, Cavaco L, Hasman H (2010). Decreased susceptibility to zinc chloride is associated with methicillin resistant *Staphylococcus aureus* CC398 in Danish swine. Vet Microbiol.

[17] Cavaco LM, Hasman H, Aarestrup FM (2011). Zinc resistance of *Staphylococcus aureus* of animal origin is strongly associated with methicillin resistance. Vet Microbiol.

[18] Mama OM, Ruiz-Ripa L, Lozano C, Gonzalez-Barrio D, Ruiz-Fons JF, Torres C (2019). High diversity of coagulase negative staphylococci species in wild boars, with low antimicrobial resistance rates but detection of relevant resistance genes. Comp Immunol Microbiol Infect Dis.

[19] Igbinosa EO, Beshiru A, Akporehe LU, Oviasogie FE, Igbinosa OO (2016). Prevalence of methicillin-resistant *Staphylococcus aureus* and other *Staphylococcus* species in raw meat samples intended for human consumption in Benin city, Nigeria: implications for public health. Int J Environ Res Public Health.

[20] O’Brien AM, Hanson BM, Farina SA (2012). MRSA in conventional and alternative retail pork products. PLoS One.

[21] Wu S, Huang J, Wu Q (2018). *Staphylococcus aureus* isolated from retail meat and meat products in China: incidence, antibiotic resistance and genetic diversity. Front Microbiol.

[22] Tulinski P, Fluit AC, Wagenaar JA, Mevius D, van de Vijver L, Duim B (2012). Methicillin-resistant coagulase-negative staphylococci on pig farms as a reservoir of heterogeneous staphylococcal cassette chromosome *mec* elements. Appl Environ Microbiol.

[23] de Freitas Guimaraes F, Nobrega DB, Richini-Pereira VB, Marson PM, de Figueiredo Pantoja JC, Langoni H (2013). Enterotoxin genes in coagulase-negative and coagulase-positive staphylococci isolated from bovine milk. J Dairy Sci.

[24] Rolo J, Worning P, Nielsen JB (2017). Evolutionary origin of the staphylococcal cassette chromosome *mec* (SCC*mec*). Antimicrob Agents Chemother.

[25] Wang YT, Lin YT, Wan TW (2019). Distribution of antibiotic resistance genes among *Staphylococcus* species isolated from ready-to-eat foods. J Food Drug Anal.

[26] Abdalrahman LS, Wells H, Fakhr MK (2015). *Staphylococcus aureus* is more prevalent in retail beef livers than in pork and other beef cuts. Pathogens.

[27] Li L, Chen Z, Guo D (2017). Nasal carriage of methicillin-resistant coagulase-negative staphylococci in healthy humans is associated with occupational pig contact in a dose-response manner. Vet Microbiol.

[28] Argudin MA, Lauzat B, Kraushaar B (2016). Heavy metal and disinfectant resistance genes among livestock-associated methicillin-resistant *Staphylococcus aureus* isolates. Vet Microbiol.

[29] Lee HH, Lee GY, Eom HS, Yang SJ (2020). Occurrence and characteristics of methicillin-resistant and -susceptible *Staphylococcus aureus* isolated from the beef production chain in Korea. Food Sci Anim Resour.

[30] Nair R, Thapaliya D, Su Y, Smith TC (2014). Resistance to zinc and cadmium in *Staphylococcus aureus* of human and animal origin. Infect Control Hosp Epidemiol.

[31] Slifierz MJ, Park J, Friendship RM, Weese JS (2014). Zinc-resistance gene *czrC* identified in methicillin-resistant *Staphylococcus hyicus* isolated from pigs with exudative epidermitis. Can Vet J.

[32] Argudin MA, Butaye P (2016). Dissemination of metal resistance genes among animal methicillin-resistant coagulase-negative staphylococci. Res Vet Sci.

[33] de Lourdes RS, da Cunha M, Calsolari RAO, Junior JPA (2007). Detection of enterotoxin and toxic shock syndrome toxin 1 genes in *Staphylococcus*, with emphasis on coagulase-negative staphylococci. Microbiol Immunol.

[34] Schelin J, Wallin-Carlquist N, Cohn MT, Lindqvist R, Barker GC (2011). The formation of *Staphylococcus aureus* enterotoxin in food environments and advances in risk assessment. Virulence.

[35] Czop JK, Bergdoll MS (1974). Staphylococcal enterotoxin synthesis during the exponential, transitional, and stationary growth phases. Infect Immun.

[36] Wang G, Wang H, Han Y (2017). Evaluation of the spoilage potential of bacteria isolated from chilled chicken *in vitro* and *in situ*. Food Microbiol.

